# The Antioxidant Potential of Tomato Plants (*Solanum lycopersicum* L.) under Nano-ZnO Treatment

**DOI:** 10.3390/ijms241411833

**Published:** 2023-07-23

**Authors:** Katarzyna Włodarczyk, Beata Smolińska, Iwona Majak

**Affiliations:** 1 Institute of Natural Products and Cosmetics, Department of Biotechnology and Food Sciences, Lodz University of Technology, ul. Stefanowskiego 2/22, 90-537 Lodz, Poland; katarzyna.wlodarczyk@dokt.p.lodz.pl; 2 Institute of Food Technology and Analysis, Department of Biotechnology and Food Sciences, Lodz University of Technology, ul. Stefanowskiego 2/22, 90-537 Lodz, Poland; iwona.majak@p.lodz.pl

**Keywords:** tomatoes, antioxidants, nanoparticles, nano-ZnO

## Abstract

Tomato (*Solanum lycopersicum* L.) is one of the most valuable horticulture crops, consumed in both its raw and processed forms. To increase yield and efficiency, conventional and organic fertilizers are utilized in modern agriculture. Traditional fertilizers increase crop yield but are harmful to the environment. These circumstances motivate the pursuit of an alternate solution. The purpose of this research was to investigate how the application of nanoparticles (nano-ZnO) combined with conventional fertilizer influence tomato plants’ development, including the antioxidant potential of cultivated plants. Three factors such as different types of cultivars, dosage of applied nano-ZnO solution and the method of nanoparticles application were implemented. Multiple analysis of selected antioxidants content and their activities such as malondialdehyde (MDA), flavonoids, polyphenols, ascorbic acid, peroxidase (POX), superoxide dismutase (SOD) or catalase (CAT) were analyzed. The obtained data exhibited that all examined parameters were strongly dependent on three implemented factors: concentration of nano-ZnO suspension, the type of cultivated tomato and the method of nanoparticles application. For instance, the accumulation of MDA in cultivated plants was different among plants under nanoparticles treatment, but in one specific case (Malinowy Bossman cultivar treated with 50 mg/L nano-ZnO suspension) the content of this marker was decreased by 34% in comparison to the corresponding control. Nevertheless, the results presented in this study showed that the usage of certain doses of nano-ZnO suspension may increase the antioxidant potential of tomato plants.

## 1. Introduction

*Solanum lycopersicum* L., commonly known as tomato, is a highly esteemed horticultural crop that contains various health-promoting constituents, including carotenoids. These carotenoids, along with other antioxidants such as lycopene, are responsible for the characteristic red color of tomatoes. In addition, it is noteworthy that fresh tomato fruit is found to contain roughly 12 mg/100 g of lycopene, alongside other carotenoids such as β-carotene, phytoene, and lutein. Several studies suggest that these health-promoting compounds may reduce the risk of cancer. Furthermore, the ingestion of tomatoes and their carotenoid constituents is advised for safeguarding against cardiovascular diseases and decreasing the likelihood of contracting other illnesses such as colon, stomach or rectal cancer [1,2,3,4]. Tomatoes are a rich source of various nutrients, including vitamins and polyphenols such as anthocyanins and phenol acids. The peel of tomatoes also contains a small amount of flavonoids [5]. The aforementioned category of substances comprises naringenin, chalcone, flavonol, rutin and quercetin glycoside. Apart of the popularity of tomato plants and the fact that those crop plants are cultivated all over the world, the increasing human population has forced the agricultural sector to increase crop productivity in order to fulfil the needs of billions of people [6]. Producing enough food for the world’s increasing population has become a serious problem. Moreover, the agriculture sector faces many problems such as finding an ecologically friendly but at the same time efficient way to grow crop plants properly. This situation may require the use of new technologies in the area of crop fertilization. The conventional bulk fertilizers are becoming less effective and have negative impacts on the environment. The increasing production efficiency, the requirements of food safety and environmental protection should be provided immediately. Nanotechnology in the form of nanofertilizers may be the solution for improving the efficiency and safety of fertilization. An example of nanomaterials that can be used in future as a potential nanofertilizer are zinc oxide nanoparticles (ZnO-NPs) which are widely used in many other sectors like medicine or electronics.

Although the utilization of nanoparticles in the agricultural industry is gaining popularity and receiving increased attention in research, there remain several aspects that necessitate further investigation. For instance, understanding the mechanism of interactions between nanoparticles and plants is vital to evaluate their positive and negative effects on agricultural processes and environment. Unfortunately, the knowledge about the abovementioned interactions is limited. To gain the required knowledge of the risks presented by nanoparticles in the environment and for the identification of biomarkers for nanoparticle toxicity, the biochemical and molecular processes of plant–nanoparticle interactions must be examined. The majority of research on the phytotoxicity of nanomaterials has relied on phenotypic characteristics, such as seed germination and root elongation, to determine the direct impacts of nanoparticles on plant metabolism [7]. Nanoparticles have diverse, complicated, and occasionally poorly understood models of action on biological structures. Several biochemical indicators, including metabolite composition, membrane integrity, and enzyme activity, have been employed to examine the influence of environmental stress on plants. The most frequently described mechanisms of nanoparticle toxicity in plants are the cell surface coating which might cause mechanical damage or clogging of pores [8,9], the increased production of reactive oxygen species (ROS) causing oxidative stress [10], and release of toxic metal ions [8,10]. Higher plants have a complex antioxidant defense mechanism which is used to counteract the accumulation of harmful reactive oxygen species (ROS). Generation of ROS may be the effect of oxidative stress which appears when plants are exposed to both biotic and abiotic stress (for instance drought, high salinity, etc.). Redox homeostasis in plants is maintained by two options of the antioxidant machinery: the enzymatic components consisting of the superoxide dismutase (SOD), ascorbate peroxidase (APX), guaiacol peroxidase (GPX), glutathione-S-transferase (GST), and catalase (CAT), and the non-enzymatic low molecular compounds such as ascorbic acid (AA) [11,12]. Numerous studies have shown that the usage of NPs may affect multiple parameters related to plants’ growth and development. The application of different NPs or their oxide forms sometimes yielded in beneficial effects in examined plants, however in certain circumstances, it is perceived by the plant as an abiotic stress factor. The knowledge on this subject is still limited, and more studies are required. Moreover, few studies addressed the molecular level interaction between nanoparticles and plants and NP-phytotoxicity. The available data indicate that smaller nanoparticles may migrate via symplast (like plasmodesmata), but bigger nanoparticles concentrate in the apoplastic space, according to physiological research. Nevertheless, the knowledge about the abovementioned interactions is still limited. In addition to existing knowledge gaps concerning the mechanism by which plants uptake nanoparticles, there remains a lack of consistent information associated to the overall response of plants to nanoparticle utilization. The available research on the impact of nanoparticles (NPs) on plant response exhibits significant variability, even when considering studies involving the same plant species and nanoparticles. Numerous studies have identified several factors that can potentially have influence on the occasionally distinct results, including the specific growing conditions employed or the precise dosage of nanoparticles applied. Therefore, there is a crucial need to further enhance and extend the scope of research associated with exposing plants to varying dosages and types of nanoparticles.

For instance, although the impacts of Zn on plants have been widely studied, fewer studies have examined the effects of nanoform of Zn on plant responses. Certain studies proved the beneficial effect of nano-Zn application on tomato growth parameters, especially the use of nano-ZnO. Nevertheless, disadvantages of those NPs were provided as well. The uptake of nanoparticles by plants is regulated by several variables, including the nature of the particles, their interaction with the environment, and the physiology of the plant [13,14,15]. Several studies were carried out in order to examine the influence of Zn NPs or nano-ZnO NPs on tomato plants. In the research conducted by Amooaghaie et al. (2016) [16], both Zn (25 nm) and ZnO (15 nm) NPs were utilized in cultivation of tomato (*Solanum lycopersicum* L.) and wheat (*Triticum aestivum* L.). At lower concentrations, Zn and ZnO NPs promoted seed germination and increased growth parameters, however at higher concentrations, NPs had the opposite effect. Nonetheless, dissolved zinc (3–23 mg/L) in the form of NPs had no significant effect on the germination parameters of either species. Compared to bulk ZnO, NPs exhibited more toxicity on seed germination and additionally reduced chlorophyll and carotenoid levels. Moreover, NPs at a certain concentration induced oxidative stress in both plants, and the variations in proline accumulation and APX activity [16]. In the research of Faizan et al. (2019) [17], the foliar application of ZnO NPs at concentration 50 ppm induced an increase in several growth features. On the 60th day of treatment the increase in several parameters was recorded in comparison with corresponding control. The obtained results indicated the increase in shoot length (30%), root length (29%), chlorophyll content (32%) or fruit yield (19%), but nano-ZnO utilization affected the antioxidant system in examined tomatoes as well. Significant increases of catalase (60%), peroxidase (74%), and superoxide dismutase (55%) activity were recorded. Moreover, in response to this treatments, lycopene (23%) and β-carotene (25%) content increased significantly, however ascorbic acid quantity decreased by 38%. In the research of Alharby et al. (2016) [18] the influence of ZnO NPs (15–30 mg/L) on tomato plants was investigated parallel with exposure to salt (3–6 g/L). Even though exposure to increasing NaCl concentration was related with growth rate inhibition and increased activity of antioxidants such as SOD (superoxide dismutase) and GPX (guaiacol peroxidase), nano-ZnO mitigated these effects. Interestingly, the lower concentration of NPs provided stronger efficacy in this case. Moreover, this study is one of the few in which the impact of NPs was evaluated on several cultivars of the same plant. The obtained results demonstrate that various tomato cultivars exhibited different degrees of resistance to salt in the presence of ZnO NPs. Important data were provided by the research conducted by Raliya et al. (2015) [19] in which tomato plants were treated with ZnO NPs in two different methods of application: foliar and soil mediated application. It was discovered that aerosol-mediated application was more successful than soil-mediated application for the absorption of nanoparticles by plants. In comparison to the control, plants treated via soil application method with the use of ZnO NPs at 250 mg/kg demonstrated a height increase of 25%. With foliar spraying, plant height increased by between 4 nd 11%. The relative chlorophyll content of tomato plant leaves was considerably increased by the application of ZnO NPs via both applications. Overall, foliar treatment was shown to promote more lycopene production than soil application.

The attempt was made to thenvestigate how the application of nanoparticles (nano-ZnO) combined with conventional fertilizer influence tomato plants’ growth and development, including the antioxidant potential of cultivated plants. An additional aspect of the research conducted was to combine the application of nano-ZnO with the use of a standard fertilizer (biohummus) to support the fertilization of plants and to compare if the usage of chosen NPs may interfere the effects of fertilizer. In addition, the innovation of this study was the analysis of the influence of three variables on the obtained results: cultivar type, dosage of applied nano-ZnO solution, and application method of nanoparticles. In this research multiple analysis of selected antioxidants content and their activities were analyzed (including the examination of malondialdehyde (MDA), flavonoids, polyphenols, and ascorbic acid content as well as the activity of peroxidase (POX), superoxide dismutase (SOD), or catalase (CAT)).

## 2. Results

The presence of selected antioxidants and their activity were analyzed in green, aboveground parts of cultivated plants. In the case of the Maskotka cultivar, the plants were either treated with ZnO NPs by soil application (SA) or via foliar spraying (FS). Two other cultivars (Granit and Malinowy Bossman (MB)) were treated with ZnO NPs by soil application. In addition, the cultivated plants were nourished with Biohumus SuperForte (except Blank sample). The research has been implemented to investigate the effect of usage of combined NPs and standard fertilizer on the plants’ nutrients absorption. A comprehensive explanation of the symbols that were applied to this part of the study are found in Table 1.

### 2.1. The Content of Malondialdehyde (MDA)

**Figure 1:**
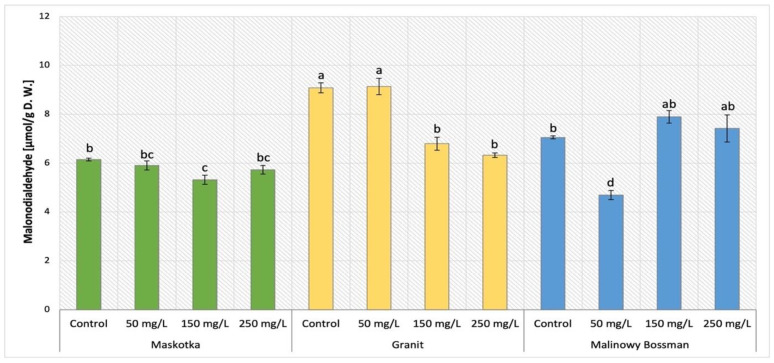
Malondialdehyde (MDA) content in tomato leaves of three cultivars. Vertical bars are used to display all data, which are the means of 3 replicates (±SD). Different letters indicate the significant differences for *p*-value ≤ 0.05.

MDA content was a commonly employed measure of lipid peroxidation in plant tissue, which increases in response to oxidative stress. This parameter was considered as an oxidative stress marker in leaves. The results obtained after MDA content analysis in cultivars treated with NPs by soil application (Maskotka, Granit and MB) were presented in Figure 1. The statistical analysis of obtained data indicates that both examined factors such as concentration of NPs and the cultivar type influence the MDA content. In the case of Maskotka plants treated with nano-ZnO by soil application, a minor reduction in MDA content was observed. For plants treated with 50 and 250 mg/L NPs suspension, the MDA content decreased by approximately 4% and 7%, respectively, compared to control. The content of MDA in Maskotka was considerably reduced in the case of SA 150, while the achieved value was lower than control (by 13%). Granit cultivar presented the opposite tendency. The highest MDA content was observed in plants treated with 50 mg/L of NPs solutions, while the usage of higher dosage of NPs decreased the MDA content considerably. The application of 150 and 250 mg/L of nano-ZnO reduced the MDA content by 25% and 30%, respectively, compared to control. The results of MB cultivar revealed that the lowest concentrated NPs suspension exhibited the most beneficial influence on plants. The usage of NPs at 50 mg/L led to a significant decrease in MDA content. Compared to the corresponding control, the MDA content in MB plants under SA 50 treatment was decreased by a significant 34%. For higher concentrated NPs suspensions, the results were different. In comparison to the corresponding control, the abovementioned treatments increased the MDA content slightly by 12% within the usage of nano-ZnO at a dose of 150 mg/L and by 5% after using nano-ZnO at a dose of 250 mg/L.

**Figure 2:**
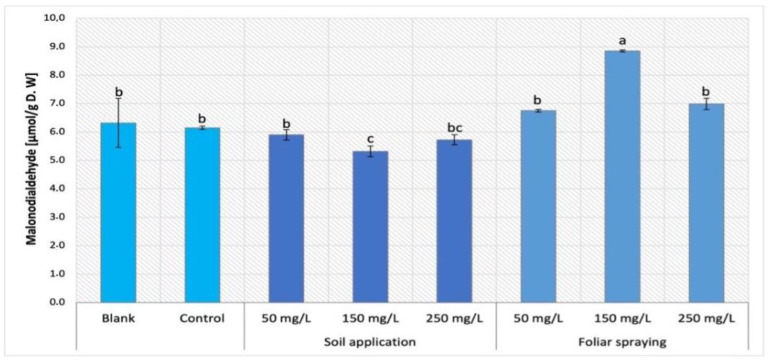
Malondialdehyde (MDA) content in tomato leaves of Maskotka cultivar. Vertical bars are used to display all data, which are the means of 3 replicates (±SD). Different letters indicate the significant differences for *p*-value ≤ 0.05.

The comparison of MDA content obtained by Maskotka plants treated with NPs via SA and FS were presented in Figure 2. The statistical analysis of obtained data for Maskotka cultivar indicates that both factors, concentration of NPs and the method of NPs application, influence the MDA content. A minor decrease in MDA content was observed in Maskotka plants treated with nano-ZnO by soil application. For plants treated with 50, 150, and 250 mg/L NPs suspension, the MDA content decreased by approximately 4%, 13%, and 7%, respectively, compared to control. Moreover, the NPs application by foliar spraying led to an increase in MDA content. The accumulation of MDA was lower for FS 50 and FS 250 when compared to FS 150. Plants treated with FS 50 and FS 250 reached an increase in MDA content by 10% and 14%, respectively, compared to control, whereas the plants sprayed with nano-ZnO at concentration of 150 mg/L had MDA content increase by 44%. Both controls (blank and control) attained a similar level of MDA content which emphasizes that the use of standard fertilizer did not affect this parameter.

### 2.2. Determination of Antioxidant Activity

**Figure 3:**
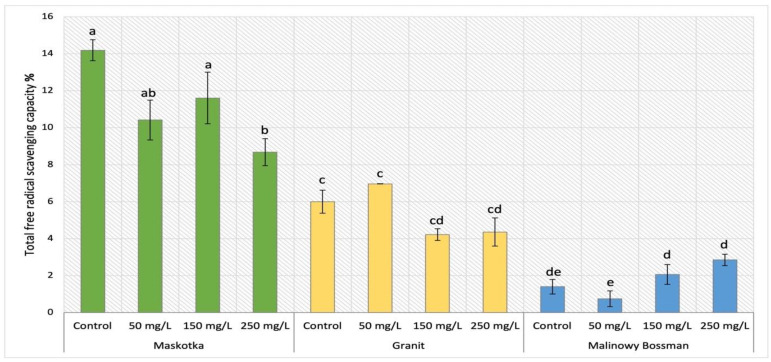
Total free radical scavenging capacity in tomato leaves of three cultivars. Vertical bars are used to display all data, which are the means of 3 replicates (±SD). Different letters indicate the significant differences for *p*-value ≤ 0.05.

The total antioxidant activity was measured by DPPH method and expressed as free radical scavenging capacity. For cultivars Maskotka, Granit and MB the results obtained after NPs treatment were demonstrated in Figure 3.

For the Maskotka cultivar a considerable decrease in free radical scavenging capacity (FRSC) was observed. This trend was correlated with the concentration of NPs suspension. For Maskotka plants under SA treatment the lowest value of FRSC was reached by plants fertilized with 250 mg/L of nano-ZnO. The obtained result displayed a decrease by 39% when compared to control. The Maskotka plants treated with lower dosage of NPs by soil application similarly exhibited the decrease, however not as significantly as was recorded for SA 250. When compared to the control, plants treated with 50 and 150 mg/L NPs reached lowered results by 27% and 18%, respectively. For the Granit cultivar the use of NPs solution led to a considerable decrease in the FRSC. Still, the plants treated with 50 mg/L suspension reached a minor increase. In comparison to the control plants, Granit SA 50 samples reached the FRSC value higher by 16%. For Granit plants under the treatment of higher dosage of nano-ZnO, a decrease in FRSC values was observed. Plants under the NPs treatment of 150 and 250 mg/L obtained FRSC significantly lower results than control (by 30% and 28%, respectively). The findings obtained by MB cultivar presented a direct correlation between the applied concentration of nano-ZnO and the increased value of FRSC. Obtained data present an exponential increase in FRSC values with the increasing concentration of NPs suspension. The usage of the lowest dosage of NPs suspension (50 mg/L) led to a significant decrease in the FRSC by 47%. However, the utilization of higher doses initiated the opposite result. Application of 150 and 250 mg/L suspension of nano-ZnO led to an increase in FRSC by 47% and 103%, respectively, compared to the corresponding control.

**Figure 4:**
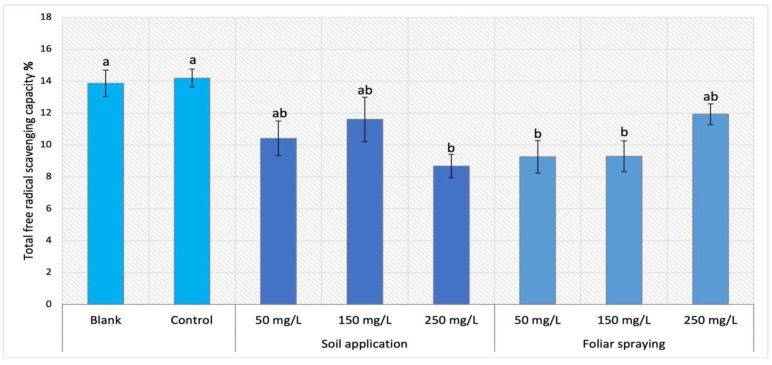
Total free radical scavenging capacity in tomato leaves of Maskotka cultivar. Vertical bars are used to display all data, which are the means of 3 replicates (±SD). Different letters indicate the significant differences for *p*-value ≤ 0.05.

For Maskotka plants under foliar spraying treatment, the trend was different than for plants under soil application treatment (Figure 4). While the SA plants reached the FRSC range between 8 and 10%, FS plants attained an outcome which varied between 9 and 12%. Subsequently, the method of NPs application was the factor influencing the FRSC values. The highest result for FS was obtained by the plants sprayed with 250 mg/L NPs suspension. Nevertheless, this value was lower than the control by 16%. For FS 50 and FS 150 the results were considerably lower, by approximately 35%, in comparison to the control sample.

### 2.3. Determination of Chlorophyll Content in Tomato Leaves

**Figure 5:**
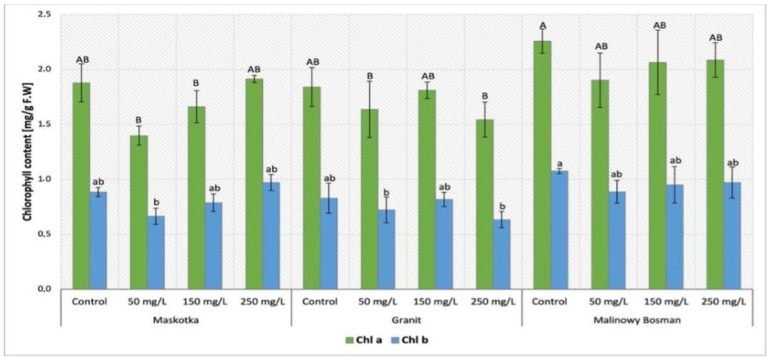
Chlorophyll (chl *a* and chl *b*) content in tomato leaves of three cultivars. Vertical bars are used to display all data, which are the means of 3 replicates (±SD). Different letters indicate the significant differences for *p*-value ≤ 0.05.

The content of selected plants’ pigments was determined in tomato leaves. Both chlorophyll (chl *a* and chl *b*) and carotenoids are the compounds that belong to plant pigments. The results of chlorophyll content analysis in plants treated with NPs via soil application (Maskotka, Granit and MB cultivars) were demonstrated in Figure 5. Results obtained by plants under the SA treatment, revealed that the factor which mainly affected the chlorophyll content was the cultivar type (*p*-value < 0.05). The results obtained from the Maskotka cultivar indicated the decrease only in plants treated with NPs suspension via soil application. A specific trend in results obtained for Maskotka plants after SA treatment was observed. The lower the concentration of NPs suspension, the lower the chlorophyll content that was detected in plants. For instance, plants treated with NP suspension at 50 mg/L reached chl *a* content reduced by 7% compared to control. For Maskotka plants treated with NPs suspension at dose 150 mg/L, the chl *a* content was decreased by 12% when compared to control. For plants supplied with nano-ZnO at a dosage of 250 mg/L the obtained value was increased by 2%. A similar trend was observed for chl *b* content. For Granit cultivar treated with 50 mg/L and 250 mg/L NPs suspension, chl *a* content was decreased by 11% and 16%, respectively, compared to the control. The same tendency was demonstrated with chl *b* content, where samples treated with 50 mg/L and 250 mg/L NPs suspension obtained results displayed a decrease by 13% and 24%, compared to the corresponding control. For MB, chl *a* content was at a similar level for all samples. The concentration of chl *a* in MB sample under NPs treatment was decreased by 19–26%, in comparison to corresponding control. Furthermore, for MB plants the chl *b* content ranged between 0.89 and 0.97 mg/g F.W. and those values were decreased by approximately 10.2–17.6% when compared to the corresponding control.

**Figure 6:**
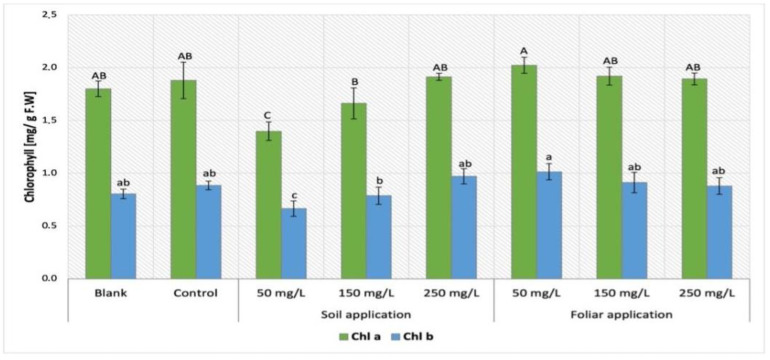
Chlorophyll (chl *a* and chl *b*) content in tomato leaves of Maskotka cultivar. Vertical bars are used to display all data, which are the means of 3 replicates (±SD). Different letters indicate the significant differences for *p*-value ≤ 0.05.

The comparison of chlorophyll content obtained by Maskotka plants treated with NPs via SA and FS were presented in Figure 6. The lower concentration of NPs suspension applied via soil, the lower chlorophyll content was exhibited by Maskotka plants. For foliar spraying of Maskotka plants, the treatment resulted in higher or comparable yields to the control. Moreover, in contrast to SA sample, higher concentrations of utilized suspension resulted in lower chlorophyll content (both chl *a* and chl *b*). The highest chlorophyll concentration was obtained by samples FS 50. Results were higher by approximately 12% (chl *a*) and 24% (chl *b*), compared to controls. With increasing concentrations of NPs suspension, the values of chlorophyll concentrations were steadily decreasing, compared to FS 50. Surprisingly, the sample treated with NPs by soil application demonstrated the opposite tendency.

### 2.4. Determination of Carotenoids Content in Tomato Leaves

**Figure 7:**
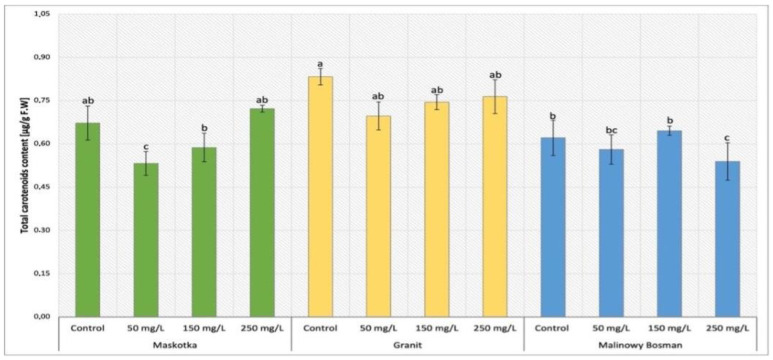
Carotenoid content in tomato leaves of three cultivars. Vertical bars are used to display all data, which are the means of 3 replicates (±SD). Different letters indicate the significant differences for *p*-value ≤ 0.05.

The analysis of carotenoids content in cultivars (Maskotka, Granit and MB) treated with nano-ZnO via soil application were demonstrated in Figure 7. The Maskotka plants treated with nano-ZnO at 50 mg/L attained the results of lowered content of carotenoids by about 21%, compared to control. The highest value reached by Maskotka plants under SA treatment was obtained by plants treated with 250 mg/L NPs suspension. The carotenoid concentration increased by over 7% when compared to the control. Furthermore, for the Granit cultivar the amount of carotenoid in plants treated with NPs was decreased when compared to control. The highest concentration of carotenoids was obtained from plants treated with 250 mg/L NPs. Nevertheless, this result displayed a decrease by 8% in comparison to the corresponding control. MB plants have revealed a different tendency to the two abovementioned cultivars. Opposite to Maskotka and Granit, the MB plants treated with 250 mg/L NPs suspension achieved the lowest concentration of carotenoids, decreased by 13% when compared to the control. Moreover, MB plants treated with suspension at 150 mg/L obtained a result slightly higher than corresponding control (by 5%).

**Figure 8:**
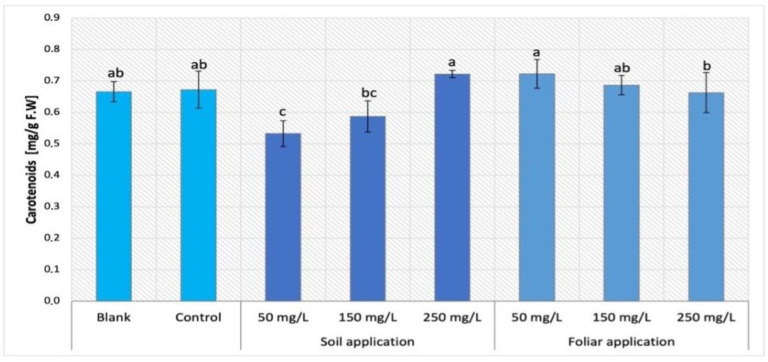
Carotenoid content in tomato leaves of Maskotka cultivar. Vertical bars are used to display all data, which are the means of 3 replicates (±SD). Different letters indicate the significant differences for *p*-value ≤ 0.05.

While the exposure to NPs via soil application resulted in decrease in carotenoid content in Maskotka plants (except SA 250), the foliar spraying provided a different trend (Figure 8). The suspension of 50 mg/L nano-ZnO appears to be the most beneficial for this parameter. The carotenoids content was increased when compared to controls by 7.5%. Interestingly, plants treated with 250 mg/L NPs via soil application and the ones sprayed with 50 mg/L NPs reached the same level of carotenoids concentration. For two other suspensions applied by FS (150 and 250 mg/L) the treatment seems to be beneficial, while the values they achieved are at the same level as the control.

### 2.5. Total Phenolic Content

**Figure 9:**
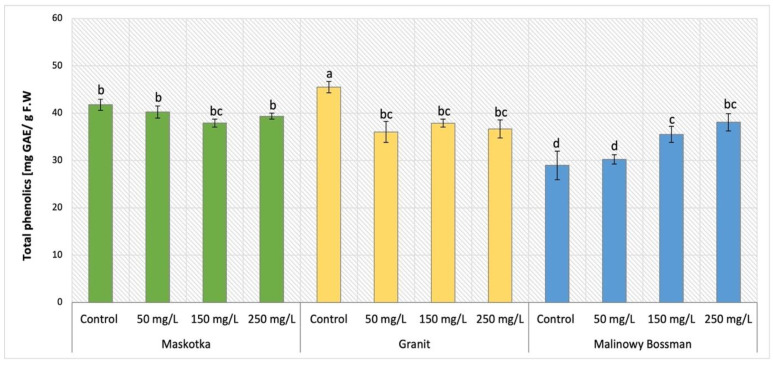
Total phenolic content in tomato leaves of three cultivars. Vertical bars are used to display all data, which are the means of 3 replicates (±SD). Different letters indicate the significant differences for *p*-value ≤ 0.05.

The content of phenolics which are the most prominent secondary metabolites in plants was evaluated in tomato aboveground parts of plants and the obtained results were demonstrated in Figure 9. The obtained data revealed common trend for two cultivars, Maskotka and Granit. For those two tomato cultivars, the applied treatment initiated a decrease in total phenolic content. For Maskotka cultivar treated with NPs via SA the observed decline in total phenolic content was milder. For samples which were treated with NPs via soil application, compared to control, the total phenolic content was reduced by approximately 4–9%. For the Granit cultivar the reduction was greater than for Maskotka plants. Application of nano-ZnO suspension at concentration of 50, 150 and 250 mg/L led to a decrease in total phenolic content by 21%, 17%, and 19% respectively. In contrast to both abovementioned cultivars, for MB the tendency of revealed results was opposite. The higher the concentration of utilized NPs suspension, the higher the concentration of total phenolics that was obtained. The MB plants treated with nano-ZnO via soil application at doses 50, 150, and 250 mg/L obtained total phenolic content increased by 4.3%, 22.6%, and 31.4%, respectively, when compared to the corresponding control.

**Figure 10:**
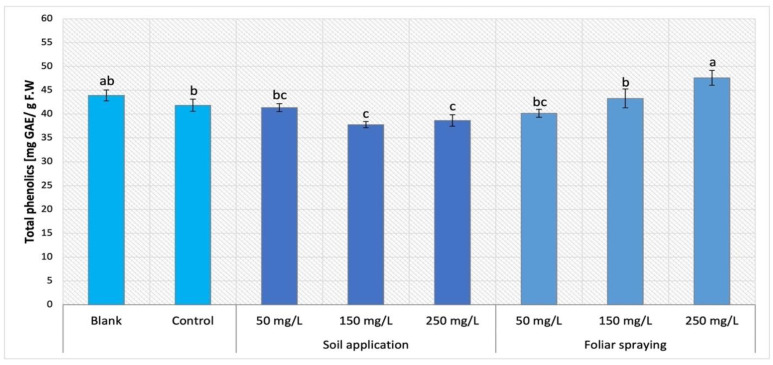
Total phenolic content in tomato leaves of Maskotka cultivar. Vertical bars are used to display all data, which are the means of 3 replicates (±SD). Different letters indicate the significant differences for *p*-value ≤ 0.05.

On the other hand, the foliar spraying treatment of Maskotka cultivar conveyed an opposite result to SA treatments. The results obtained by Maskotka plants treated with NPs by both SA and FS method were presented in Figure 10. While the soil application method led to a decline in the total phenolic content, the FS mostly caused an increase in the amount of those compounds. For FS, higher concentration of NPs allows to obtain higher result. The use of FS at dosage 150 and 250 mg/L led to an increase of 3.3% and 13.6%, respectively when compared to the corresponding control. Solely, foliar-sprayed samples with 50 mg/L NPs suspension obtained slightly decreased results in comparison to the control (by 2.9%).

### 2.6. Total Flavonoid Content

**Figure 11:**
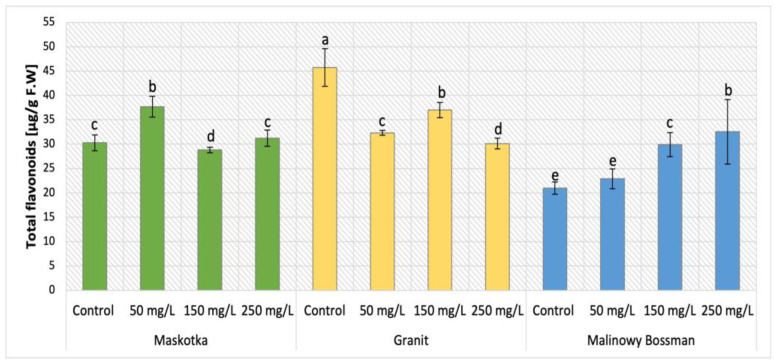
Total flavonoid content in tomato leaves of three cultivars. Vertical bars are used to display all data, which are the means of 3 replicates (±SD). Different letters indicate the significant differences for *p*-value ≤ 0.05.

Among phenolic secondary metabolites, flavonoids are exceptionally abundant. The analysis of total flavonoid content in cultivars (Maskotka, Granit, and MB) treated with nano-ZnO via soil application was demonstrated in Figure 11. The examination of total flavonoid content in all three tomato cultivars revealed that both factors, type of cultivar and dosage of used NP suspension, affect the final concentration. For Maskotka plants the nano-ZnO displayed results which vary significantly when compared to control. For instance, the usage of NPs at 50 mg/L affected an increase in flavonoid content, by 24%, compared to the corresponding control. Nevertheless, plants exposed to higher concentrations of soil applied NPs led to a decrease in flavonoids. In comparison to the control, the NPs suspension at dose 150 mg/L caused a decrease in flavonoid content by approximately 5%, while the use of 250 mg/L nano-ZnO suspension caused a minor increase in flavonoid content. The Granit cultivar showed opposite results. All implemented dosages of used NPs suspension caused a decrease in flavonoid concentration in plants. Samples treated with nano-ZnO at concentration of 50, 150, and 250 mg/L reached values decreased by 29%, 19%, and 30%, respectively, when compared to the control. For the MB cultivar it was noted that as the concentration of the utilized NPs solution increased, the concentration of detected flavonoids increased. Both dosages of NPs, 150 mg/L and 250 mg/L led to increase by 42% and 55% respectively, in comparison to control. The lowest dosage of NPs resulted in minor increase as well.

**Figure 12:**
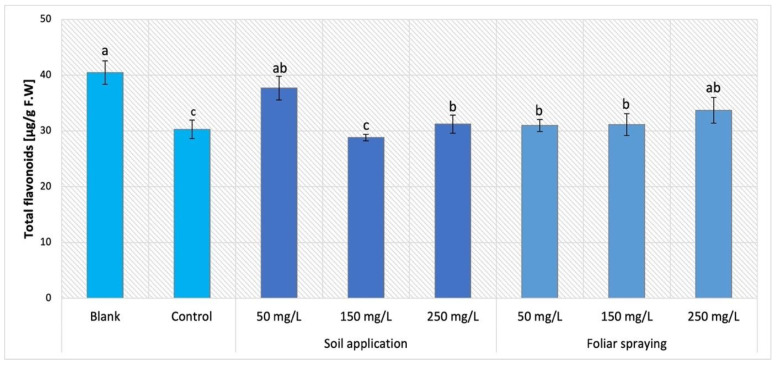
Total flavonoid content in tomato leaves of Maskotka cultivar. Vertical bars are used to display all data, which are the means of 3 replicates (±SD). Different letters indicate the significant differences for *p*-value ≤ 0.05.

The results of NPs influence applied by SA and FS on Maskotka plants were demonstrated in Figure 12. Foliar NPs spraying application for Maskotka plants initiated minor increase in flavonoids content in comparison to the control. Nevertheless, the level of flavonoids was increased by 2–11%.

### 2.7. Ascorbic Acid Content

**Figure 13:**
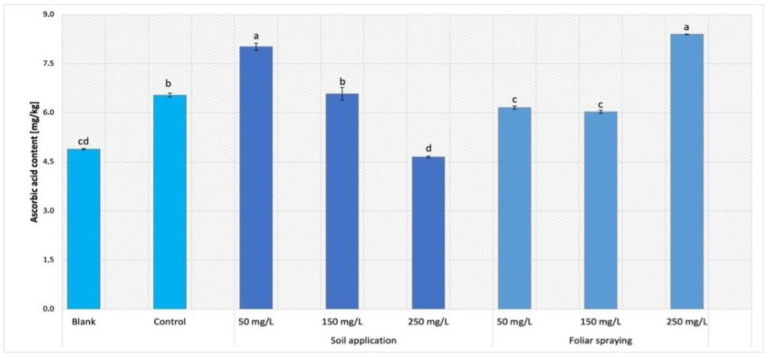
Ascorbic acid content in tomato leaves of Maskotka cultivar. Vertical bars are used to display all data, which are the means of 3 replicates (±SD). Different letters indicate the significant differences for *p*-value ≤ 0.05.

The content of ascorbic acid was examined only in leaves of Maskotka plants (Figure 13). For this parameter, both implemented factors, method of NPs application and the concentration of the used suspension, affected results significantly. For SA samples, a concentration of 50 mg/L allowed highest result to be obtained. The amount of ascorbic acid in plants treated with the abovementioned dosage of NPs was raised by approximately 23% compared to control. Nevertheless, nano-ZnO suspension at 150 mg/L led to obtaining the ascorbic acid content similar to control. In comparison to the control, the usage of suspensions at dose of 250 mg/L decreased the vitamin C concentration by almost 29%. Foliar spraying treatment caused the opposite results when compared to SA method. The samples exposed to 50 and 150 mg/L NPs solution have reached values slightly lower than the control sample. However, the plants exposed to the highest concentration of NPs achieved the highest value of vitamin C. The obtained value was equal to 8.4 mg/kg, which displays an increase of 28% compared to control. The blank sample (except SA 250) achieved the lowest value of vitamin concentration.

### 2.8. Activity of Pyrogallol Peroxidase (POX)

**Figure 14:**
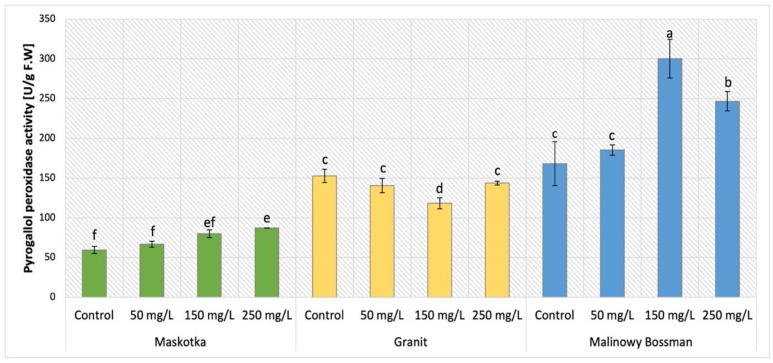
Pyrogallol peroxidase (POX) activity in tomato leaves of three cultivars. Vertical bars are used to display all data, which are the means of 3 replicates (±SD). Different letters indicate the significant differences for *p*-value ≤ 0.05.

The activity of pyrogallol peroxidase (POX) was evaluated in plants treated with NPs (cultivars Maskotka, Granit, and MB) by soil application and the obtained results are presented in Figure 14. The statistical analysis indicated that both implemented factors (NPs concentration and method of its application) strongly influenced the results. Maskotka plants under SA treatment displayed an increase in POX activity. The application of 50 mg/L NPs suspension did not considerably affect the POX activity, however the usage of higher doses (150 and 250 mg/L) led to a minor increase in POX. In comparison to the control, the NPs at doses 150 and 250 mg/L led to an increase in POX activity by 35% and 47%, respectively. For Granit plants NPs suspension at all applied doses led to a decrease in POX activity. The lowest result was obtained by plants treated with NPs at dose of 150 mg/L. The achieved result was decreased by 22.4%, compared to the corresponding control. The POX activity detected in plants treated with nano-ZnO at doses of 50 and 250 mg/L was comparable and slightly declined in comparison to control (by 7%). For the MB cultivar, all applied NP solutions caused an increase in POX activity. The highest POX activity in MB plants was observed in samples treated with 150 mg/L nano-ZnO via soil. The results obtained from plants under the abovementioned treatment were considerably elevated when compared to the corresponding control (by 79%). MB plants exposed to 250 mg/L achieved an increase in POX activity as well, when compared to the control (by 47%).

**Figure 15:**
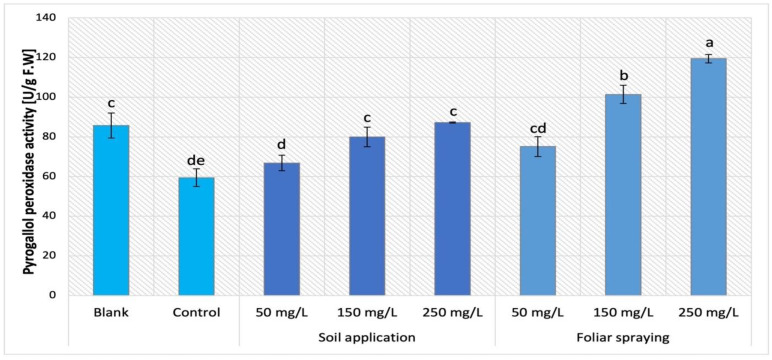
Pyrogallol peroxidase (POX) activity in tomato leaves of Maskotka cultivar. Vertical bars are used to display all data, which are the means of 3 replicates (±SD). Different letters indicate the significant differences for *p*-value ≤ 0.05.

The supplementation of nano-ZnO in both forms, SA and FS, affected the POX activity in Maskotka plants, which is demonstrated in Figure 15. Maskotka cultivar displayed a similar trend to MB, either by plants enriched with NPs via soil application or via foliar spraying. Yet, a more significant increase in activity was noted for plants under foliar spraying treatment. The increase presented by Maskotka plants under FS treatment was linear. The highest result was obtained by plants treated with nano-ZnO at dose of 250 mg/L, the activity of POX was increased by 101% when compared to the control. Moreover, plants treated via foliar spraying with 50 and 150 mg/L displayed significant increase in POX activity by 26% and 71%, respectively, compared to the control.

### 2.9. Activity of Superoxide Dismutase (SOD)

**Figure 16:**
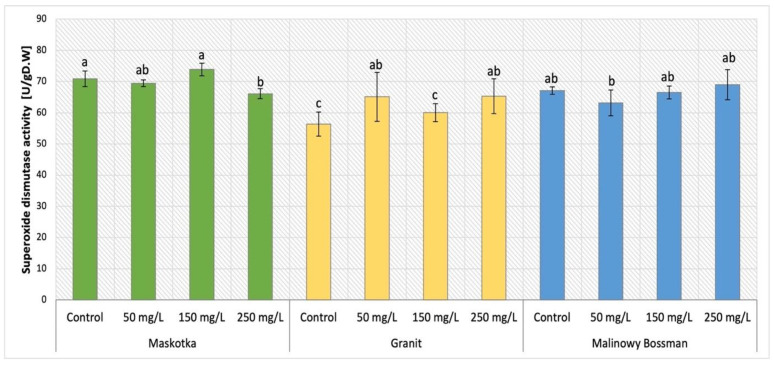
Superoxide dismutase (SOD) activity in tomato leaves of three cultivars. Vertical bars are used to display all data, which are the means of 3 replicates (±SD). Different letters indicate the significant differences for *p*-value ≤ 0.05.

The analysis of SOD activity in all three tomato cultivars supplemented with nano-ZnO via soil application was demonstrated in Figure 16. The analysis of obtained results indicate that the type of cultivar was the main factor that affects this parameter. The activity of SOD presented by Maskotka cultivar (under SA treatment) exposed the specific tendency. The soil application of NPs suspension at 150 mg/L led to achieving the highest value of SOD activity in plant tissues. Nevertheless, the difference between this sample and the control was minor, while for SA 150 the SOD activity increased by only 4%. The plants treated with 50 and 250 mg/L NPs suspension displayed inferior SOD activity, although those values were lower than control by less (approximately) than 5% and 7% respectively. In the case of the Granit cultivar, the SOD activity was slightly higher in plants under NP treatment than in corresponding control. However, the differences between those values are not significant. Plants treated with NPs at 50 and 250 mg/L reached results higher than control by approximately 16%. For MB cultivar the obtained activity of SOD did not vary significantly among plants. For this cultivar the use of NPs did not considerably affect the SOD activity in plants’ tissues.

**Figure 17:**
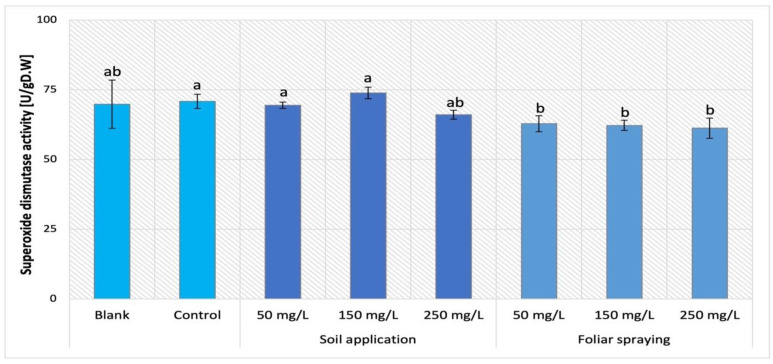
Superoxide dismutase (SOD) activity in tomato leaves of Maskotka cultivar. Vertical bars are used to display all data, which are the means of 3 replicates (±SD). Different letters indicate the significant differences for *p*-value ≤ 0.05.

The supplementation of nano-ZnO in both forms, SA and FS, had an influence on the POX activity in Maskotka plants. The obtained data for this cultivar were demonstrated in Figure 17. The values of SOD activity presented by Maskotka plants under foliar spraying treatment were even lower than the one obtained by plants treated by soil application. All applied dosage of foliar spraying led to similar results. The SOD activity exhibited by those plants decreased by approximately 11–14% when compared to control.

### 2.10. Activity of Catalase (CAT)

**Figure 18:**
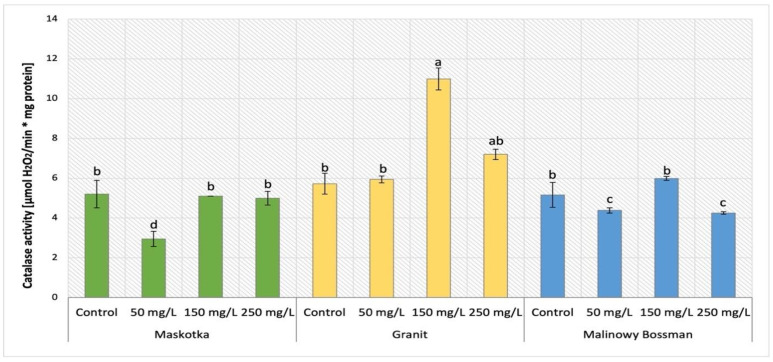
The analysis and comparison of the catalase activity in tomato leaves of three cultivars. Vertical bars are used to display all data, which are the means of 3 replicates (±SD). Different letters indicate the significant differences for *p*-value ≤ 0.05.

The analysis of catalase (CAT) activity in tomato plants (Maskotka, Granit and MB) under NP treatment applied via soil was demonstrated in Figure 18. The statistical analysis indicated that the main factor which affects this activity was the concentration of the utilized NP solution. CAT activity obtained by controls of all three cultivars reached a similar value equal to 5.2 ± 0.3 [μmol H_2_O_2_/min·mg of protein]. For the Maskotka cultivar the usage of NPs via soil application generally did not affect the CAT activity. The exception were plants treated with 50 mg/L suspension. For this application, the CAT activity was decreased by a significant 43%. For the Granit cultivar, the application of 150 mg/L NPs solution led to achieving the highest result, while CAT activity obtained for those plants were increased by 16% when compared to control. The lowest dose of NPs (50 mg/L) did not affect the CAT activity in Granit plants while the highest dose increased this activity significantly. The result achieved by the latter was raised by 26%, in comparison to corresponding control. For MB the soil application of 150 mg/L NPs solution led to achieving the highest result, while CAT activity obtained for those plants was higher by 92%. For MB cultivar the other treatments (NPs at conc. 50 and 250 mg/L) caused the CAT activity to decrease considerably, both by approximately 16%, in comparison to the corresponding control.

**Figure 19:**
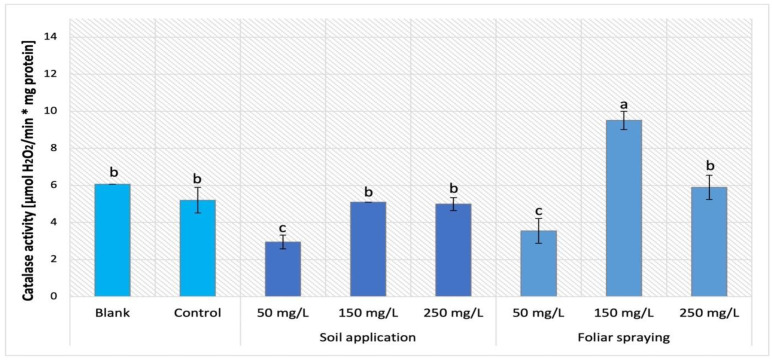
The analysis of catalase activity in tomato leaves of Maskotka cultivar. Vertical bars are used to display all data, which are the means of 3 replicates (±SD). Different letters indicate the significant differences for *p*-value ≤ 0.05.

The influence of NPs treatment via SA and FS application on Maskotka plants is presented in Figure 19. Similarly, as was observed for the Maskotka SA 50 plant, the CAT activity in the FS 50 samples was decreased by 32% when compared to the control. Moreover, the FS samples treated with higher dosage of NPs presented a considerably different outcome. Plants sprayed with 150 mg/L of nano-ZnO displayed the highest result, which was 82.9% higher than control. The usage of the highest concentration of NPs led to obtaining a CAT activity similar to the one achieved by control. Either blank or control presented similar CAT activity.

## 3. Discussion

### 3.1. The Content of Malondialdehyde (MDA)

Malondialdehyde (MDA) is a compound generated by membrane lipids in reaction to reactive oxygen species and it is an oxidative stress marker (ROS). MDA content is a commonly employed measure of lipid peroxidation in plant tissue, which increases in response to oxidative stress. This parameter is considered as an oxidative stress marker in leaves. Subsequently, the analysis of MDA content in tomato plants under NPs treatment allowed to observe if the nano-ZnO application could be considered as stressor for cultivated plants.

The data obtained indicate that NPs dosage, method of NPs application, and the type of cultivar significantly affect the accumulation of MDA in plants tissues. Firstly, the method of NP application is the factor that influenced the MDA accumulation in Maskotka plant tissues. The comparison of MDA content in plants treated via soil application and foliar spraying led to a conclusion that the latter is less beneficial for plants. The Maskotka plants under SA treatment achieved a concentration of MDA lower or similar to the values obtained by control or even blank samples. On the contrary, the foliar spraying with nano-ZnO led to an increase in MDA content in plant tissue, especially when sprayed with 150 mg/L of NPs. The two other factors which affected the MDA content were the type of cultivar and the utilized concentration of nano-ZnO suspension. For aforementioned Maskotka plants, soil application of NPs caused a decrease in MDA concentrations, while for two other examined cultivars the tendency in obtained results was different. For Granit cultivar, the usage of 150 and 250 mg/L led to a considerable decrease in MDA content. Similarly for MB cultivar the dosage of 50 mg/L of NPs causes a significant reduction in MDA accumulation.

The examination of MDA content in tomato plants under nano-ZnO treatment was performed in the study of Amooaghaie, Norouzi, and Saeri (2016) [16]. In this research the exposure to nano-ZnO suspension of 100–200 mg/L significantly increased the MDA content in tomato. Surprisingly, the authors presented limited data of this analysis since only the results of plants treated with suspensions at concentration of 100 and 200 mg/L were presented. The lack of results obtained for plants treated with other dosages of NPs hindered the full analysis. Nevertheless, the differences between the results from study of Amooaghaie, Norouzi, and Saeri (2016) [16] and this research could be the effect of several discrepancies between the two studies, such as dried plant material used for MDA analysis and the amount of Zn2+ delivered with NPs solutions. Even though in both studies the concentration of applied NPs suspensions was similar, the volume of applied solutions during cultivations and consequently the amount of provided Zn content was different. Overall, the available data suggest that the use of nano-ZnO affects the same plants differently when the conditions of cultivation vary significantly from each other.

### 3.2. Determination of Antioxidant Activity

The total antioxidant activity in cultivated plants was measured with the use of DPPH method and expressed as free radical scavenging capacity (FRSC). The analysis of FRSC can serve as a reliable indicator of the plant’s comprehensive antioxidant activity. The application of NPs may result in two potential outcomes if there is an observed increase in antioxidant activity. The initial hypothesis asserts that the observed rise in antioxidant activity within the plant is indicative of the potential damage caused by nano-ZnO, as the application of nanoparticles is perceived by the plant as a stressor. The second theoretical consideration of the observed phenomenon promotes the utilization of NPs. Plants exhibiting elevated levels of antioxidant activity might have improved resistance to external stressors, for instance for pathogenic attack.

The analysis of antioxidant activity brought similar observations to those after MDA content analysis. The obtained data indicated that examined factors like NP dosage or the method of its application significantly affect the antioxidants activity in tomato leaves tissues. For Maskotka plants, regardless of the method of nano-ZnO application, the NP treatment caused a decrease in FRSC, when compared to the corresponding control. A similar trend was observed for Granit plants with exception of samples after soil application of nano-ZnO at a dosage of 50 mg/L, while those plants achieved FRSC comparable to the control. In the case of FRSC analysis, the MB cultivar had increased values in tissues of plants under SA of NPs at doses 150–250 mg/L. Those findings were similar to the results presented in the study of Ahmed et al. (2023) [20] in which tomato plants were foliar sprayed with suspensions of nano-Zn or nano-ZnO. In the case of DPPH assay the noticeable increase in scavenging activity was observed in plants treated with nano-ZnO at doses 75, 100, and 125 ppm (mg/L) and plants treated with nano-Zn at a dose of 1500 ppm (mg/L). The comparison of the findings from research of Ahmed et al. (2023) [20] with those provided in this research may be only conducted for Maskotka plants under foliar spray treatment. In contrast to findings presented in work of Ahmed et al. (2023) [20], for Maskotka plants the implementation of NPs caused a decrease in FRSC. The differences between those could be a result of the amounts of delivered Zn ions to plants or the fact that DPPH assays in those two studies were conducted on different tomato cultivars. Overall, the same observation was made in this research, though all examined cultivars reacted differently on the exposure to NPs. Nevertheless, the fact that the usage of nano-ZnO affect the antioxidant activity could be confirmed in the research conducted by Pérez-Labrada et al. (2019) [21]. In their study tomato plants were grown under greenhouse conditions and treated with copper NPs via foliar spraying (at dose 250 mg/L (25 mL)). In addition, a group of plants treated with Cu NPs were under salinity stress. The analysis of antioxidant activity by DPPH showed no significant differences between treated plants and control what may suggest that in contrast to ZnO NPs, the usage of other nanoparticles like Cu NPs do not influence the antioxidant activity.

### 3.3. Chlorophyll and Plant Pigments Content

Chlorophylls and carotenoids are compounds that belong to plant pigments which absorb light energy to conduct the process of photosynthesis. In higher plants, two major chlorophyll (Chl) pigments (Chl *a* and Chl *b*) can be found, as well as hundreds of carotenoids (Car). The pigment content of plants is species-specific and varies with the season and leaf age. Moreover, the pigment content is influenced by environmental factors.

Chlorophyll is the most important compound in plants, though it is necessary in photosynthesis. Photosynthesis is a process by which plants convert solar energy into biochemical energy, which is then used to sustain the vast majority of life on Earth. The growth of plants is dependent on photosynthesis and therefore weakening or interrupting this process by for instance disruption of chlorophyll biosynthesis would have fatal consequences.

#### 3.3.1. Determination of Chlorophyll Content in Tomato Leaves

The determination of chlorophylls in the tomato leaves tissues disclosed the same pattern for all cultivars. The plants under NPs treatments showed a decrease in chlorophyll content (both chl *a* and chl *b*). Furthermore, the evaluation of this parameter and statistical analysis indicated that the method of NPs application was the factor that influenced the chlorophyll content the most. In contrast, Maskotka plants treated with nano-ZnO via soil application had lowered chlorophyll concentration, while the foliar spraying treatment did not affect this parameter considerably or led to a minor increase in chlorophyll content. This observation may suggest that implementation of ZnO NPs through soil could affect the processes related with chlorophyll synthesis (down-regulation of genes encoding chlorophyll synthesis). It should be noted that, during the foliar spraying, only the aboveground parts of plants are directly exposed to NPs. Moreover, chlorophyll is already synthetized inside plant tissues, thus contact with NPs does not affect its amount. The decrease in chlorophyll content in tomato plants under nano-ZnO treatment was also observed in the research of Wang et al. (2018) [22]. Similar to the presented research, tomato plants in the study of Wang et al. (2018) [22] were enriched with nano-ZnO through the soil application at doses 200, 400, and 800 mg/L. Lower concentration of chlorophylls was detected in plants treated with nano-ZnO at concentration of 800 mg/L which led to 60–70% reductions in chl *a* and chl *b* content, respectively. Nonetheless, the chlorophyll content in plants treated with lower doses was not noticeably different from controls. In addition, the yellow leaf color was observed on the leaves treated with NPs at 800 mg/L. Subsequently, for those leaves the expressions of chlorophyll synthesis genes were reduced. In the research of Faizan et al. (2021) [23] the foliar application of ZnO NPs (10, 50 and 100 mg/L) substantially increased the chlorophyll content by 13%, 34%, and 19%, respectively, when compared to control plants. Furthermore, the treatment of ZnO NPs effectively alleviates the adverse impacts induced by NaCl on diverse photosynthetic parameters. Comparable data were obtained in this research, while the highest chlorophyll concentration was obtained by plants foliar sprayed with NPs at a dose of 50 mg/L and it was approximately higher by 12% (chl *a*) and 24% (chl *b*) compared to the controls. With increasing concentrations of NPs suspension, the values of chlorophyll concentrations were staidly decreasing, compared to FS 50, but chlorophyll content in those plants was still higher than in control.

The research of Raliya et al. (2015) [19] showed the opposite results. The application of ZnO nanoparticles through foliar spray and soil delivery resulted in a significant increase in the relative chlorophyll content of the leaves of 28-day-old tomato plants. Plants that received comparable treatment with ZnO nanoparticles exhibited the highest chlorophyll content at a concentration of 750 mg/kg, while plants treated with foliar spraying demonstrated a maximum increase during exposure to concentration of 1000 mg/kg. Due to large disparities between studies, such as utilization of different features of nanoparticles, varying dosages, and application methods, the fundamental mechanism responsible for the impact of nanoparticles on photosynthetic pigments remains an unresolved issue.

#### 3.3.2. Determination of Carotenoids Content in Tomato Leaves

The analysis of carotenoids content showed the adverse impact of NPs on tomato cultivation. Almost all applied dosages of nano-ZnO led to a decrease in carotenoids in plant tissues. A few samples were an exception, as they showed an increase in carotenoids, but the differences between controls and increased carotenoids content were insignificant. In the case of Maskotka plants the application of NPs by soil at dose 250 mg/L and by foliar spraying at dose 50 mg/L caused a slight increase in carotenoids concentrations. For two other cultivars the exposure to nano-ZnO led to a decline of carotenoid concentration. Similar observations were made in the research of Amooaghaie, Norouzi, and Saeri (2016) [16] where all nano-ZnO treatment (except 50 mg/L) considerably reduced the total carotenoid content, when compared to the control. A dosage of 50 mg/L as bulk ZnO increased the photosynthetic pigments in both examined plants, wheat and tomato, when compared to control. Chl *a*, chl *b* and carotenoids concentrations in wheat were not significantly reduced, while in tomato tissues the decrease in chl a, chl b, and carotenoid content began at 100 and 200 mg/L of bulk ZnO, respectively. Overall, NPs exhibited a more significant inhibitory effect on photosynthetic pigments than ZnO in bulk. In contrast, the content of photosynthetic pigments decreased significantly and progressively as the Zn^2+^ concentration in solutions increased. On the other hand, the study of Wang et al. (2018) [22] contradicted the abovementioned findings. The tomato plants exposed to highly concentrated NP suspensions (400 and 800 mg/L) achieved a significant increase in carotenoid concentration. Moreover, the ratio of carotenoids to chlorophylls increased in plants subjected to ZnO NPs, with an eight-fold increase in plants exposed to 800 mg/L of ZnO NPs. In addition, expressions of carotenoid synthesis genes, such as PSY and LYCB, increased, though to varying degrees. The differences in tendencies of carotenoids content which were observed between the study of Wang et al. (2018) [22] and this research could be due to applied doses of nano-ZnO. In this research the utilized suspensions were at concentrations of 50, 150, and 250 mg/L, which during the whole time of cultivation delivered 9, 28, and 46 mg of Zn. In the study of Wang et al. (2018) [22] the used NP suspensions were provided at doses 200, 400, and 800 mg/L, but the authors did not provide the information about the concrete amount of Zn that was delivered to plants. This lack of information impeded the detailed comparison of results obtained in those two studies. Nevertheless, the examination of carotenoid content conducted by Amooaghaie, Norouzi, and Saeri (2016) [16] helped to draw a conclusion that the usage of nano-ZnO in tomatoes cultivation may lead to a decrease in carotenoid content in leaves’ tissues if they are applied at specific dosage. This phenomenon may occur due to the influence of NPs on the genes encoding the synthesis of carotenoids.

### 3.4. The Non-Enzymatic Antioxidant Defense System

Both abiotic and biotic stress caused the reactive oxygen species (ROS) accumulation in plants tissues which causes rapid cell injury. Plants’ cells have evolved a complex system of enzymatic and non-enzymatic antioxidant defense systems that assist in the removal of these endogenously produced ROS. A plant’s non-enzymatic antioxidant defense system consists of compounds such as ascorbic acid, tocopherols, glutathione, phenolics, or flavonoids, all of which play a crucial role during abiotic stress. Antioxidants that are non-enzymatic interact with essential physiochemical processes in plants and induce tolerance to abiotic stress [24].

Phenolics are the most prominent secondary metabolites in plants, and their distribution is visible throughout the entirety of metabolic processes. The phenolic substances, also known as polyphenols, are composed of numerous types of compounds, including simple flavonoids, phenolic acids, complex flavonoids, and colored anthocyanins. Typically, phenolic compounds are associated with defense responses in plants. Due to their antioxidant activity, they are essential compounds for plants’ defense [24].

The analysis of total phenolic content in plants’ leaves showed that all three examined factors such as method of NPs delivery, the utilized doses of NPs, and the type of examined tomato cultivar affected the phenolic content in plants tissues. Firstly, the method of NPs application was the factor which strongly influenced the obtained results. This observation was well illustrated by the results of phenolic analysis in the Maskotka cultivar. Increasing concentration of utilized NPs caused the decrease in total phenolic content in tissues of Maskotka plants treated via soil application. The opposite tendency was observed in Maskotka plants under the treatment of foliar spraying. The other factors influencing the phenolic content in plants were the NPs dosage and the type of grown tomato cultivar. Subsequently, in the case of the Granit cultivar, regardless of the concentration of used nano-ZnO, the phenolic content was decreased in comparison to the control. For MB cultivar the increasing dosage of NPs led to an increase in phenolic content. Unfortunately, to the best of author’s knowledge, only a few studies were carried out in which the effect of nano-ZnO on the phenolic content in tomato was determined. Those studies were mostly focused on the phenolic content in tomato fruits and not in leaves like in this research. Nonetheless, in the study of Pérez-Labrada et al. (2019) [21] tomato plants were treated with copper (Cu) NPs via foliar spraying (with dose of 250 mg/L) and additionally some plants were kept under salinity stress. Total phenolic content in tomato leaves was increased by all treatments compared to the control, but highest values were obtained when Cu NPs were applied. The foliar spaying of Cu NPs on tomato plants under saline conditions increased the total phenolic content by 5% compared to plants stressed with NaCl but lacking Cu NPs. Identical results were observed in the fruits [21]. The changes in the total phenolic content can be related to the changes of individual phenolic compounds in the leaves of tomato exposed to heavy metals such as Cu or Zn. The content of total phenolic may increase or decrease in various plants under the abiotic stress which may be caused by the exposition to NPs. Additionally, in the research of Ahmad et al. (2020) [25] the exposure to nano-ZnO affected the antioxidants content in leaves of candy leaf (*Stevia rebaudiana* L.). A significant increase in total phenolic content was observed for plants treated with 2 mg/L of nano-ZnO, while the usage of higher doses (20, 200 or 2000 mg/L) led to a decline of total phenolic content. The comparison of results obtained in research of Ahmad et al. (2020) [25] and with analysis of total phenolic content in this research is impossible due to multiple differences between those two studies. Nevertheless, the example of the study of Ahmad et al. (2020) [25] showed that the implementation of ZnO NPs may have a beneficial impact on the phenolic content in different types of plants. The effect of applied NPs strongly depends on the utilized dosage and the conditions of cultivation.

Among phenolic secondary metabolites, flavonoids are extremely abundant. They are mostly found as yellow pigments in plant leaves and flowers, with a lesser frequency in fruit and wood, and occasionally in seeds. Flavonoids are phytoalexins, substances with a defensive function, formed when a plant meets a pathogen [24].

As flavonoids are part of the phenol family, similar results would be expected in analysis of total phenolic content. Mostly, the results of total flavonoids analysis were comparable to the ones obtained in phenolic content examination, though with some exceptions. In comparison to the control, Maskotka plants treated with NPs through soil had decreased concentrations of flavonoids for treatment of nano-ZnO in dosages of 150 or 250 mg/L. The highest concentration of flavonoids was obtained by plants treated with NPs at 50 mg/L, whereas those plants had an increased content of flavonoids in comparison to the corresponding control. This is the most noticeable difference between the results presented in total phenolic analysis and total flavonoids analysis. It can be assumed that, for this tomato cultivar, the nano-ZnO at dose 50 mg/L directly affected the flavonoids among the phenolic compounds, while the higher doses did not influence this parameter. This observation led to the conclusion that the certain dose of Zn ions applied to plants through soil directly affect the mechanism of flavonoids synthesis. On the other hand, certain NP doses (depending on the examined cultivar) may inhibit the flavonoids accumulation in plants. Nonetheless, the more detailed analysis (i.e., the analysis of expression of genes encoding flavonoid synthesis) should be conducted to further explain this topic. Maskotka plants under foliar spraying treatment showed an increase in flavonoid content. For the Granit cultivar, regardless of the concentration of used nano-ZnO, the flavonoid content was decreased in comparison to the control. For MB cultivar the increasing dosage of NPs led to an increase in phenolic content. In the research of Pérez-Labrada et al. (2019) [21] the foliar spraying of tomatoes with Cu NPs revealed that the administration of Cu NPs increased flavonoid content by 9% in plants under salinity stress, when compared to the plants without NPs. The application of Cu NPs also led to an increase in the flavonoid content in the fruits, whereas the concentration of flavonoids in plants grown under salinity conditions (with or without the application of Cu NPs) caused a decrease by an average of 13% compared to the control treatment [21]. When comparing those findings to the results obtained in this study it can be assumed that the implementation of NPs such as Cu or ZnO has a beneficial effect on the flavonoids content in tomatoes, though the important factor that should be further examined is the concentration of chosen NPs suspensions adapted to specific plants. In the study of Ahmad et al. (2020) [25] the exposure to nano-ZnO affected the flavonoids content in leaves of candy leaf (*Stevia rebaudiana* L.). Similarly, as was observed with phenolic content, the total flavonoids content was increased for candy leaf (*Stevia rebaudiana* L.) plants under treatment of 2 mg/L of nano-ZnO. The usage of higher doses (20, 200 or 2000 mg/L) led to a decline of flavonoids content. Apart from the fact that in abovementioned study of Ahmad et al. (2020) [25] different plants were exposed to nano-ZnO than tomatoes, the gathered data helped to draw a conclusion that in general NPs have the ability to affect the flavonoids accumulation in leaves tissues.

Vitamin C (ascorbic acid (AA)) is an essential compound for plants. Ascorbic acid serves as a significant redox buffer and cofactor for enzymes involved in regulating photosynthesis, hormone biosynthesis, and the regeneration of other antioxidants [24].

The analysis of AA content in examined tomato plants was conducted in plant leaves. The presented results vary significantly depending on both implemented factors, method of NPs application, and the concentration of the used suspension. The examination of AA concentration was carried out only in Maskotka plants, though the impact of two variables on the AA accumulation was analyzed, namely, the dosage of supplemented NPs suspensions and the method of its application. Both investigated factors affect the AA concentration in plant tissues. All plants under NPs treatment reached a higher level of AA concentration than blank samples. This phenomenon indicated that the use of fertilizer may have affected the presence of ascorbic acid. Nevertheless, the additional usage of nano-ZnO combined with the standard fertilizer influenced the content of ascorbic acid in tomato leaves. Interestingly, the concentration of AA in plant tissues strongly depended on the method of application. Divergent patterns were noted between SA and FS plants, whereby an increase in the concentration of NPs suspension resulted in a reduction of AA levels in SA plants. Conversely, an increase in the concentration of utilized nanoparticle suspension resulted in an associated increase in the concentration of AA detected in the FS plants.

To the best knowledge of the authors, there is a deficiency of research in which the NPs influence on the AA content in plants was evaluated. However, most of them were focusing on the AA content in fruits and not in leaves. In the study of Faizan et al. (2019) [17] the foliar spraying with nano-ZnO (10, 50, 100 and 200 ppm) was conducted on tomato plants. While the usage of NPs led to an increase in such parameters as plant pigments, lycopene, or b-carotene, the ascorbic acid content was the only parameter which was decreased in plants under NP treatment. The usage of suspension at dose 50 ppm was the most optimal treatment, whereas it caused a significant increase in lycopene or b-carotene, and at the same time caused decrease in AA by a considerable 38% [17]. However, there was a study conducted by Li et al. (2020) [26] on the influence of Se NPs on celery (*Apium graveolens* L.) cultivation. The Se NPs suspension was applied at dose 5 mg/L via foliar spraying for 10 days. The exposure to nano-Se caused an increase in several parameters such as total antioxidant capacity (by 47%), total flavonoids (by 50%), total phenols (by 21%) and AA levels (by 27%) in celery leaves [26]. The influence of nano-ZnO on the vitamin C content in tomatoes fruits was examined in the research of Ahmad et al. (2020) [25] where the utilization of ZnO NPs caused an increase in AA content in fruits when compared to the control. All applied NPs doses of 75, 100 and 125 ppm were delivered by foliar spraying. The highest amount of ascorbic acid (22.1 mg/100 g) was recorded for treatment of 100 ppm ZnO NPs followed by the usage of 125 ppm ZnO NPs. Interestingly, those findings are opposite to the data provided by Faizan et al. (2019) [17], even though both authors were using similar doses of nano-ZnO and they were both exanimating tomatoes. However, it is important to note that in one of the studies the fruits were analyzed, while in the second research the AA was determined in plant leaves. Apart from the study of Faizan et al. (2019) [17], in two other abovementioned studies the use of NPs (Se NPs and ZnO NPs) caused an increase in AA in different plants.

Plants typically act against stress by producing compounds that can neutralize reactive oxygen species (ROS), such as non-enzymatic and enzymatic antioxidants. In this context, ascorbic acid (AA) is one of the ubiquitous non-enzymatic antioxidants capable of not only scavenging ROS, but also modulating several fundamental functions in plants under both stress and non-stress conditions [24]. The data gathered in the abovementioned studies of Li et al. (2020) [26] and Ahmad et al. (2020) [25], as well as the findings from this study, confirmed that the specific usage of NPs during plant cultivation can lead to an increase in AA accumulation in plants tissues. Subsequently, the higher concentration of AA in plant tissues can induce damage to plant tissues when they are exposed to some stressors (both biotic and abiotic).

### 3.5. The Enzymatic Antioxidant Defense System

Higher plants possess an advanced antioxidant defense system that serves to mitigate the accumulation of damaging reactive oxygen species (ROS). The production of reactive oxygen species (ROS) may be a consequence of oxidative stress, which manifests in plants when they are subjected to various biotic and abiotic stressors, such as drought and high salinity. Plants maintain redox homeostasis through two distinct options of the antioxidant machinery. These options include enzymatic components such as superoxide dismutase (SOD), ascorbate peroxidase (APX), guaiacol peroxidase (GPX), glutathione-S-transferase (GST), and catalase (CAT) [27].

Peroxidase (POX) is a member of a group of hemoproteins with a highly variable structural composition. These enzymes facilitate the redox reaction between hydrogen peroxide and certain reductants. Peroxidases are involved in multiple cellular processes in plants, including growth and stress responses. In fact, they regulate growth by regulating hormones and cell wall metabolism as well as the antioxidant defense. Therefore, these enzymes are considered biomarkers for biotic and abiotic stresses [24,27].

The analysis of POX activity in tomato leaves brought varying results which were strongly dependent on the type of cultivar that was taken under consideration. For Maskotka plants, for both methods of NPs application, the increasing doses of supplemented nano-ZnO were related to increasing POX activity. Contrary observations were made for the Granit cultivar, while all implemented doses of NPs caused a decrease in POX activity. For the MB cultivar, in general, the application of NPs through soil caused an increase in POX activity, though only the use of 150 and 250 mg/L doses caused a significant increase in POX activity, when compared to the control. The beneficial influence of nano-ZnO on POX activity in tomato plants was also reported in research of Faizan et al. (2019) [17]. The highest peroxidase activity was observed in plants foliar sprayed with nano-ZnO at doses 50 ppm (68% measured at 45th day of plants cultivation and 75% of measured at 60th day of plants cultivation). Further analysis of nano-ZnO impact on tomato plants, conducted by Faizan et al. (2021) [23] brought similar observations. Moreover, foliar application of nano-ZnO at 50 mg/L enhanced the POX activity (by 59%) at plants under salinity stress. Those findings confirmed that nano-ZnO has a direct influence on the POX activity in tomato plants, while the foliar treatment of ZnO-NPs increased the performance of antioxidative enzymes in presence and absence of NaCl [23]. In contrast to the studies mentioned, in the research of Amooaghaie, Norouzi, and Saeri (2016) [16] the exposure of tomato seedlings and plants to the presence of Zn nanoforms did not affect the POX activity. In multiple studies it has been reported that the exposure to NPs affects the activities of antioxidant enzymes in plants, indicating that the degree and type of response varies with plant species, examined parts of plants, and the intensity, length, and type of NP treatment. Although the effects of NPs on plants have been extensively studied, fewer studies have examined the effects of nanoparticle size or compared the cultivation environment in which treated with NPs plant exist. Still, the data presented in multiple articles are inconsistent.

To develop crop plants that can tolerate abiotic stresses, it is now necessary to understand the plant responses to particular stresses. Superoxide dismutase serves as the first barrier against reactive oxygen species (ROS). The upregulation of SODs may help plants to survive in a stressful environment [24,27].

The evaluation of SOD activity in leaves tissues showed that application of nano-ZnO has a minor, if any, impact on this parameter. The results of SOD activity analysis in Maskotka tomato plants showed the significance of utilized method of NPs delivery. The usage of foliar spraying method caused a minor decrease in SOD activity, while in the case of soil application method, SOD activity was comparable to the control samples. Granit was the only tomato cultivar in this research in which the exposure to NPs caused an increase in SOD activity (with the use of 50 and 250 mg/L doses). For MB cultivar the usage of 50 mg/L dosage was less beneficial, though this treatment caused a noticeable decline of SOD activity. The use of higher doses of NPs led to obtaining SOD activity comparable to control. A similar trend was observed in the research of Amooaghaie, Norouzi, and Saeri (2016) [16] where the usage of nano-ZnO was applied with nutrient solution. The application of nano-ZnO at concentrations of 100 and 200 mg/L considerably increased SOD activity in tomato tissues. Moreover, in the study of Faizan et al. (2019) [17] the foliar spraying of tomato plants with nano-ZnO at doses 50 and 100 ppm increased the SOD activity by approximately 55%. Furthermore, Wang et al. (2018) [22] conducted a study in which tomato plants were treated with nano-ZnO through soil. The analysis of selected antioxidants analysis showed that ZnO NP treatment enhanced SOD activity in tomato plants in a concentration-dependent manner, although 200 mg/L ZnO NPs had barely any effect on these activities. Subsequently, the more detailed analysis demonstrated that elevated concentrations of ZnO nanoparticles resulted in an up-regulation of SOD activity, as evidenced by an increase in transcription of the Cu/Zn2-SOD and Fe-SOD genes [22].

In general, multiple studies along with this research confirmed that the exposure of tomato plants on the nano-ZnO affected the activity of SOD. In most cases the application of NPs caused the increase in SOD activity, which can be considered as a beneficial effect of such a treatment. SOD is considered to be essential for the process of detoxifying ROS in plants. The role of SOD is deemed essential in the regulation of the concentration of superoxide anion radical [28].

Environmental stressors like ultraviolet (UV) radiation or pathogens or other stressors can generate fast fluctuations of hydrogen peroxide levels, which leads to oxidative stress. Catalase (CAT) is the predominant H_2_O_2_-scavenging enzyme and an important element of plants defense which degrades H_2_O_2_ into water and oxygen [24,27].

The factor which affected the CAT activity in examined tomato plant was the concentration of utilized suspension. For Maskotka plants the NPs dose of 50 mg/L caused a significant decrease in CAT activity, either in plants treated via soil application or in plants foliar sprayed. The higher doses of NPs implemented in soil applied to Maskotka plants led to CAT activity at comparable level to the control. For Maskotka plants under FS treatment usage of 150 mg/L, NPs caused a considerable increase in CAT activity. Similar observations were made for Granit plants. For MB plants the usage of NPs at a dose of 150 mg/L enhanced CAT activity, but not so significantly as in the case of two other cultivars. Faizan et al. (2019) [17] reported the positive impact of nano-ZnO on CAT activity in tomato plants. The plants that were subjected to foliar spraying with nano-ZnO at a dosage of 50 ppm exhibited a higher increase in CAT activity (60%). Furthermore, in another study Faizan et al. (2021) [23] conducted additional analysis on the effect of nano-ZnO on tomato plants, yielding comparable findings. In addition, the application of nano-ZnO at a concentration of 50 mg/L resulted in a significant increase in catalase activity (by 57%) in plants subjected to salinity stress. The results indicated that nano-ZnO has a significant impact on CAT activity in tomato plants. Furthermore, the application of ZnO NPs via foliar treatment has been found to enhance the performance of antioxidative enzymes in the presence and absence of NaCl [23]. The beneficial impact of ZnO NPs on tomato growth and antioxidants activity was reported by Wang et al. (2018) [22]. The authors presented an analysis which results indicated that tomato treated with nano-ZnO had enhanced CAT activity in leaf tissues. Additionally, the higher expression of *CAT1* (gene which enables CAT activity) was observed in plants exposed to high concentrations of ZnO NPs [22].

## 4. Materials and Methods

For the research on nano-ZnO influence on tomato plants, three cultivars of cherry-type tomato (*Solanum lycopersicum*) cultivars were chosen; Maskotka (W. Legutko), Granit (Plantico), and Malinowy Bossman (Plantico). All seeds of chosen cultivars are commercially available. The fruits of all three cultivars displayed similarities. 

In the prepared plastic pots containing soil, previously sterilized seeds were sown (each cultivar separately) and then hydrated. Before sowing, seeds were sterilized by exposure for 1–2 s to 70% ethanol and then placed in a beaker with 30% ACE (sodium hydrochloride) for 30 min. Next, after exposure to ethanol seeds were rinsed with distilled water 4 times [29] and then placed in the pots with soil. After five days of incubation in the dark, the containers were placed on a 12/12 day/night cycle at 25 ± 3 °C (air humidity approximately 50%). After a further week of incubation, individual sprouting was transferred to 150 g of soil in separated container. Next, 50 mL of fertilizer (Biohumus SuperForte, Agrecol J.P., Mesznary, Poland) and 30 mL of ZnO NPs (<50 nm) solution were added. The suspensions of NPs at concentrations of 50 mg/L, 150 mg/L, and 250 mg/L were used. In addition, two types of control samples for Maskotka plants were cultivated simultaneously. The blank sample contained neither fertilizer nor nanoparticle solutions, while the control contained only fertilizer. For two other cultivars, Granit and Malinowy Bossman, only one type of control sample was prepared, the pot with corresponding seed which was enriched only with fertilizer.

**Figure 20:**
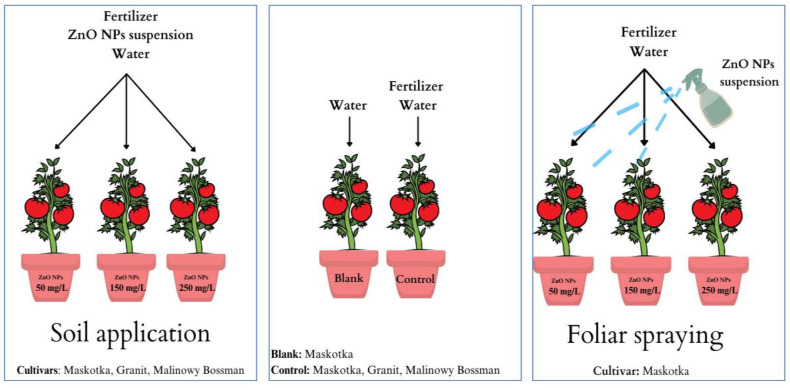
The graphic illustration of the scheme of plants’ treatments.

After one month of germination, each sprouting was transferred into new individual containers containing 350 g of fresh soil (150 g of soil from previously used pots and additional portion of 200 g fresh soil). There were three replicates of each treatment. For the Maskotka cultivar, two methods of NP solution application were utilized: soil treatment and foliar spraying. Only soil treatment was used for cultivars Granit and Malinowy Bossman (Figure 20). For plants intended for soil application, NP solutions of a specific concentration were applied shortly after seeding in new containers. The first foliar application of NP’s on Maskotka plants was provided two weeks after transferer to new containers. Selected plants were sprayed with the NPs solution every two weeks, and all plants were fertilized (50 mL) and treated with the NP solution approximately once per month (30 mL). All plants were cultivated for 6 months and after that period leaves of grown plants were collected and used for analysis.

ZnO NPs suspensions were prepared of <50 nm (Sigma Aldrich, Saint Louis, MO, USA). Suspension of ZnO NPs was prepared with varying concentrations (50, 150, 250 mg/L) in deionized water and dispersed by ultrasonic vibration (100 W, 40 kHz) for 30 min to avoid aggregation (Bandelin Sonorex DT 102 H). The characteristics of ZnO NPs are demonstrated according to the reports of Sigma Aldrich company (Table 2). In the work of Rajput et al. (2021) [30], the physical and chemical characteristics of the nanoparticles used in this investigation were extensively examined.

### 4.1. The Content of Malonodialdehyde (MDA)

To determine malondialdehyde (MDA) content in leaves, the 2-thiobarbituric acid (TBA) in accordance to the method of Hodges et al. (1999) [31] was applied. The first step of the procedure was to ground the 1 g of plant tissues with 5 mL 0.6% TBA in 10% trichloroacetic acid (TCA). Subsequently, mixture was heated at 100 °C for 15 min and then cooled in ice.

Lastly, samples were centrifuged at 5000 rpm/min for 10 min. The absorbance was measured at 450, 532, and 600 nm. Data were calculated as nmol per gram of dry weight (nmol/g D.W.). The MDA content was determined on a dry weight basis as follows:

MDA (μmol/g D.W.) = 6.45(A_532_– − A_600_)– − 0.56A_450_

### 4.2. Determination of Antioxidant Activity (DPPH)

First, antioxidants were extracted from the samples by mixing 2 mL of methanol with 0.05 g of grounded leaves tissue [32]. The mixtures were then placed in an ultrasonic bath for five min at 27 °C, then kept at 4 °C for 48 h. The samples were then centrifuged for 10 min at 16,000× *g*. (Centrifuge type Sigma 2-16P, Polygen, Gliwice, Poland). After two days of extract incubation, 300 mM of DPPH solution was prepared through dilution in 80% methanol (*v*/*v*). Then, 100 μL of plant extract was added to 1 mL of DPPH solution and 3.9 mL of 80% methanol. The absorbance was measured after 5, 15, and 30 min of incubation against blank at 517 nm. All analyses were performed in triplicate. The DPPH radical scavenging activity was calculated from the following equation:

RSC [%] = 100 (A_0_– − A)/A_0_

where:

A—average absorbance of the sample.

A_0_—average absorbance of a control (DPPH).

### 4.3. Chlorophyll and Plant Pigments Concentration

Determination of plant pigments was performed following the procedure described by Israelstam and Hiscox (1979) [33] with the modifications of Richardson et al. (2002) [34]. Tomato leaves were mixed with 3 mL of pre-heated at 65 °C dimethylsulfoxide (DMSO) solution. Next, samples were incubated in water bath (65 °C) for 30 min. Following the incubations, samples were centrifuged and the measurement of absorbance (UV/VIS8453 Spectroquant Nova 400 spectrophotometer (Merck kGaA, Darmstadt, Germany)), was conducted. The measurements were taken for four different wavelengths: 663 nm, 645 nm, 470 nm, and 534 nm. The content of individual plant dyes was calculated according to the formula of Richardson et al. (2002) [34]. The results were given in mg/g fresh weight of the plant.

### 4.4. Total Flavonoid Content

The total flavonoid content in tomato leaves was determined using the aluminum chloride colorimetric method, with slight modification [35]. As a standard for this reaction, the quercetin was used, and the results were expressed as mg of quercetin per 1 g fresh weight of the plant (mg QE/g F.W.). The plant tissue was homogenized with 80% ethanol, mixed for one hour and then centrifuged. Next, the extract solution (0.5 mL) was mixed with 1.5 mL of 80% methanol, 0.1 mL of 10% aluminum chloride hexahydrate (AlCl_3_), 0.1 mL of 1 M potassium acetate (CH3COOK), and 2.8 mL of deionized water. After incubation at room temperature for 30 min, absorbance of the reaction mixture was measured at 415 nm [36] (UV/VIS 8453 Spectroquant Nova 400 spectrophotometer (Merck kGaA, Darmstadt, Germany)).

### 4.5. Total Phenolic Content

The total content of phenolic compounds was measured in accordance to the Folin–Ciocalteu method [37]. The plant extracts were prepared by homogenization of plant tissue with 80% methanol. Samples were incubated in room temperature for 30 min and then centrifuged. Following, 0.5 mL of extracted samples were mixed with Folin–Ciocalteu reagent and incubated for five min. Next, 4 mL of Na_2_CO_3_ (1 M) was added and the mixture was left at room temperature for 15 min. The content of phenolics was determined by a spectrophotometer at 765 nm (UV/VIS8453 Spectroquant Nova 400 spectrophotometer (Merck kGaA, Darmstadt, Germany)). The total phenolic content in samples was described as the amount of gallic acid equivalent per gram of fresh weight of the plant (mg GAE/g F.W.).

### 4.6. Ascorbic Acid Content

The profile of ascorbic acid in tomato leaves was analyzed by the UHPLC–ESI–MS technique. The leaf extracts were prepared by mixing leaves with chloroform. Next, extracts were dissolved in ultrapure water (20 mg/mL) and filtered through a 0.20 μm nylon syringe filter. Chromatographic analysis was conducted using a CBM-20A controller, two LC-2020AD pumps, SIL-30AC auto sampler and CTO-20AC column oven (Shimadzu, Tokyo, Japan), equipped with a photodiode array detector (SPD-M20A Prominence diode array detector, Shimadzu, Tokyo, Japan) and a mass spectrometer (LCMS-2020, Shimadzu, Tokyo, Japan), equipped with electrospray ionization source—ESI. The chromatographic separation was conducted using Kinetex C18 (150 mm × 2.1 mm × 5.0 μm) analytical column from Thermo Scientific (Waltham, MA, USA). The mobile phase solvent A consisted of methanol: formic acid (99.9:0.1, *v*/*v*) and solvent B water: formic acid (99.9:0.1, *v*/*v*) (solvent B), and a flow rate of 0.3 mL/min was used. The injection volume was 2 μL. The used gradient elution started at 5% B, reaching 75% B at 2 min, 95% B at 2.5 min, then isocratic elution was maintained for one minute. Next, gradient elution was restored to 5% at four minutes and held for 2 min. The calibration curves were constructed for standard compound using six different concentration levels in a range of 0.01–1.0 mg/mL. MS spectra were obtained in collision-induced dissociation (CID) mode using nitrogen. Instrument control, data acquisition and evaluation were performed with the LabSolutions 5.60 SP2. Chromatography Data System, and Postrun Analysis software, respectively. The mass spectrometric conditions were as follows: capillary voltage of 4.5 kV, drying gas temperature of 250 °C, drying gas flow of 15.0 L/min, and nebulizing gas pressure of 1 bar.thee column temperature was 30 °C, the capillary temperature was 350 °C, and nitrogen was used as nebulizer. Full scan mass spectra were acquired over a mass range from *m*/*z* 50 to 550 in the negative ion mode. Identification and peak assignment of ascorbic acid was based on the comparison of their retention times, UV–vis spectra characteristics and full scan mass spectra with those of authentic standards analyzed under identical conditions.

### 4.7. The Activity of Pyrogallol Peroxidase (POX)

The activity of pyrogallol peroxidase (POX) (EC. 1.11.1.7) was determined by the pyrogallol oxidation method in the presence of H_2_O_2_ in accordance with methodology of Chance and Maehly (1955) [38]. A unit of enzyme activity (U) was defined as the amount of oxidation of pyrogallol into purpurogallin at pH 6 in 20 s. The plant leaves were homogenized with the use of acetate buffer (pH 5.6), then mixed on a shaker for 30 min. The amount of 0.32 mL pyrogallol (5%, *w*/*v*), 0.16 mL H_2_O_2_ (0.5%, *v*/*v*), 0.32 mL acetate buffer (pH 5.6) and 2.2 mL distilled water were added to the tube. The reaction solution was mixed. Then 0.05 mL of plants extract was added and the whole solution was poured into a colorimetric cuvette. The absorbance of sample was recorded immediately at 420 nm for 5 min (UV/VIS8453 Spectroquant Nova 400 spectrophotometer (Merck KGaA, Darmstadt, Germany)). The enzyme activity was calculated as peroxidase activity (unit/g D.W.) [39].

$$Peroxidase\;activity\left[\frac{U}{g}D.W.\right]=\frac{\left[(sample–OD-control\Delta OD)\times 3\times df\times V\right]}{12\times 0.05\times W}$$

ΔOD—the difference of absorbance at 420 nm per 20 s over the 5 min period;

3—the volume of reaction system;

df—dilution factor;

V—the total volume of enzyme solution extracted from the material;

12—the extinction coefficient of 1.0 mg/mL purpurogallin at 420 nm;

0.05—the volume of enzyme solution added to the reaction;

W—dry weight of material used for extraction.

### 4.8. The Activity of Superoxide Dismutase (SOD)

The activity of superoxide dismutase (SOD) (1.15.1.1) was estimated following the Roth and Gilbert [40] description. The 0.5 g of plant tissues were homogenized with the use of 5 mL of extraction buffer (containing 0.1 M phosphate buffer (pH 7.5) and 0.5 mM EDTA). The enzyme extract was centrifuged for 20 min at 15,000× *g* at 4 °C. The reaction mixture contained 20 μL enzyme extract, 50 mM sodium phosphate buffer (pH 7.8), 100 μM EDTA, and 10 mM of pyrogallol. The enzyme activity was calculated by measuring the absorbance change at 420 nm (UV/VIS8453 Spectroquant Nova 400 spectrophotometer (Merck KGaA, Darmstadt, Germany)) for 120 s against a blank sample without extract. One unit of enzyme activity was taken as the amount of enzyme, which reduced the absorbance reading to 50% in comparison with tubes lacking enzyme.

$$\%\mathrm{Inhibition}\;\mathrm{of}\;\mathrm{pyrogallol}\;\mathrm{oxidation}=\frac{{∆A}_{p}}{{∆A}_{k}}\times 100\%$$

$$\mathrm{SOD}\;\mathrm{activity}=\frac{\%\mathrm{inhibition}\mathrm{of}\mathrm{pyrogallol}\mathrm{oxidation}}{2}$$

### 4.9. The Activity of Catalase (CAT)

The activity of catalase (CAT) (EC. 1.11.1.6) was assayed by measuring the disappearance of H_2_O_2_. Then, 0.5 mL of 75 mM H_2_O_2_ was added in 1.5 mL of 0.1 M phosphate buffer (pH 7) and 50 μL of diluted enzyme extract in 3 mL reaction mixture. The extract was prepared with the procedure described in 4.7. The decrease in absorbance at 240 nm was observed for one min and enzyme activity was computed by calculating the amount of H_2_O_2_ decomposed [41].

### 4.10. Statistical Analysis

The data were expressed as means ± standard deviation and analyzed statistically with the use of R Studio. The statistical significance of the treatments was evaluated by two or three factor analysis of variances (ANOVA), performed on the analyzed parameters. Analysis of variance was performed to determine the significance of differences between the pairs of means in different variants of the conducted experiment. The differences were statistically significant when *p*-value was less than 0.05, according to Tukey or Dunn statistical tests, where different letters assigned to means designate a statistical difference at *p*-value ≤ 0.05. The chosen data were investigated via non-parametric statistics on the basis of results obtained by descriptive analysis; the means were compared by the Kruskal–Wallis test, and that highlighted significant differences.

## 5. Conclusions

The maintenance of balance between the formation and degradation of reactive oxygen species (ROS) is crucial in preventing oxidative damage. Within the context of plant biology, reactive oxygen species (ROS) are effectively eliminated through the utilization of ROS-scavenging antioxidative enzymes, including superoxide dismutase (SOD), peroxidase (POX), or catalase (CAT). The increase in activity of the mentioned enzymes, as well as the content of the compounds of non-enzymatic antioxidant defense system (flavonoids, phenols, or carotenoids), may help plants to fight against the stress which may occur when plants are exposed to many factors such as drought, high salinity, pathogen attack, or other [24]. The results of non-enzymatic antioxidant defense system in tomato plants showed that nano-ZnO treatment may affect some of the compounds as was noticed in the examination of ascorbic acid content. All plants under NP treatment reached a higher level of AA concentration than blank samples. Moreover, the concentration of AA in plants tissues strongly depended on the method of application, which suggests that the selection of factors such as dosage but also the method of NPs application is crucial. Interestingly, similar observation was made in the case of other parameters. For instance, the examination of the content of chlorophylls and the evaluation of this parameter and statistical analysis revealed that the application method of NPs had the greatest impact on this parameter. In contrast, Maskotka plants treated with nano-ZnO via soil application had a lower chlorophyll concentration, whereas foliar spraying did not significantly impact this parameter and led to a slight increase in chlorophyll content. The results of enzymatic antioxidant defense system in tomato plants under nano-ZnO treatment showed that the use of nano-ZnO influenced mainly the activity of POX and CAT. The SOD activity in all examined tomato cultivars was not significantly affected by NPs usage when compared to the corresponding control. This phenomenon could be due to the fact that SOD in plants’ cells may occur in three isoforms: chloroplastic and cytosolic Cu/Zn-SOD isoform, chloroplastic Fe-SOD isoform, and Mn-SOD isoform found in the mitochondria [24]. All these enzymes catalyze the same reaction, converting the oxygen radical into molecular oxygen and hydrogen peroxide [42]. It is possible that the additional amount of Zn delivered with NPs suspension caused the vast majority of the SOD enzyme occurring in examined plants to be in the isoform Zn-SOD. On the other hand, the activity of POX or CAT was affected by the nano-ZnO application. For both enzymes, their activity was significantly changed at specific doses of NPs. It was observed that the changes of POX or CAT activities were not similar for all three cultivars. That led to a conclusion that ZnO NPs may be considered as stressors for plants at specific doses while the activity of abovementioned enzymes was increased. Nevertheless, the potential damaging effects of NPs was not visually seen, which may suggest that the usage of NPs increased the activity of chosen antioxidants, but that phenomenon could be beneficial for plants. The increased activity of POX or CAT could help plants to fight against the stressors.

The results obtained from the conducted analyses indicate that nano-ZnO nanoparticles may be a promising compound for enhancing the development of *Solanum lycopersicum* L. The increased concentration of selected elements in the studied plants indicates that the use of nano-ZnO together with fertilizer in plant cultivation had the beneficial effect of nano-ZnO on the activity of selected antioxidants. Depending on the concentration of nanoparticles used or the method of their application, the use of nano-ZnO resulted in an increase in the activity of selected antioxidants and, consequently, enhanced defense potential of plants against external factors (biotic and abiotic stressors). However, it should be noted that the effect of nano-ZnO on the plant depended not only on the nanoparticle concentration used, the application method, but also on the plant species and cultivar. The obtained results differed significantly between the varieties studied, indicating that the presence of nanoparticles in fertilization and plant cultivation requires an individual approach.

## Figures and Tables

**Table 1:** Explanation and description of the symbols used in the text.

Symbol	Explanation
Blank	Soil without fertilizer (no NPs added) used for cultivation
Control	Soil with fertilizer (no NPs added) used for cultivation
SA 50	Soil with fertilizer + application of NPs (50 mg/L) via soil
SA 150	Soil with fertilizer + application of NPs (150 mg/L) via soil
SA 250	Soil with fertilizer + application of NPs (250 mg/L) via soil
FS 50	Soil with fertilizer + application of NPs (50 mg/L) via foliar spraying
FS 150	Soil with fertilizer + application of NPs (150 mg/L) via foliar spraying
FS 250	Soil with fertilizer + application of NPs (250 mg/L) via foliar spraying

**Table 2:** Characteristics of ZnO NPs used for the experiments based on the information provided by producent.

Particle	Size [nm]	Purity [%]	Surface Area [m^2^/g]
nano-ZnO	<50 nm	97.0	>10.8

## Data Availability

Not applicable.
